# The role of data imbalance bias in the prediction of protein stability change upon mutation

**DOI:** 10.1371/journal.pone.0283727

**Published:** 2023-03-30

**Authors:** Jianwen Fang

**Affiliations:** Division of Cancer Treatment and Diagnosis, Computational & Systems Biology Branch, Biometric Research Program, National Cancer Institute, Rockville, MD, United States of America; University of Colorado Anschutz Medical Campus, UNITED STATES

## Abstract

There is a controversy over what causes the low robustness of some programs for predicting protein stability change upon mutation. Some researchers suggested that low-quality data and insufficiently informative features are the primary reasons, while others attributed the problem largely to a bias caused by data imbalance as there are more destabilizing mutations than stabilizing ones. In this study, a simple approach was developed to construct a balanced dataset that was then conjugated with a leave-one-protein-out approach to illustrate that the bias may not be the primary reason for poor performance. A balanced dataset with some seemly good conventional n-fold CV results should not be used as a proof that a model for predicting protein stability change upon mutations is robust. Thus, some of the existing algorithms need to be re-examined before any practical applications. Also, more emphasis should be put on obtaining high quality and quantity of data and features in future research.

## Introduction

The ability to predict protein stability change upon mutation is both theoretically important and practically relevant [1,2]. Consequently, many tools using machine learning (ML) technologies have been developed for that purpose in the past decades [2–13]. Recently, however, re-evaluation of some of these tools has shown inferior performance compared to the original publications and therefore low robustness [5,6,14–16]. While some researchers have suggested that low-quality data and insufficiently informative features are the primary reasons for the weak robustness of the tested algorithms [5,15–19], others have put more emphasis on a bias caused by data imbalance [3,6,20–24].

There are more destabilizing mutations than stabilizing mutations in the experimental data, resulting in an unbalanced dataset whereas a dominating group has more cases than a minor group of fewer cases. On the contrary, a balanced dataset has similar cases in different groups. Predictive models built on an unbalanced dataset usually deliver better performance for the dominating group (i.e., destabilizing mutations), than the minor group (i.e., stabilizing mutations). To overcome the data imbalance problem, hypothetical reverse mutations (HRMs), relative to experimental mutations (termed forward mutations in the following section), were utilized to achieve balanced datasets by combining native mutations with HRMs [3,12,13,22]. HRMs can be easily generated based on a physical principle that the following relation must hold: ΔΔG_AB_ = = −ΔΔG_BA_ (where ΔΔG_AB_ is the free energy change upon mutation, A and B are proteins before and after mutation).

The bias theory has its merits and the tactics of using HRMs in the training may help improve prediction performance to some degree; it falls short, however, to adequately explain why some models completely failed to predict HRMs, rather than reduced performance when HRMs were not included in the training [15]. In addition, many features used to build models are rather rudimentary and unlikely sufficiently informative for the purpose [15]. Besides, recently Yang et al. reported that less than 30% of the ProTherm [25], the database many predictive tools were developed upon, was deemed to be useful [5]. Similarly, other researchers have also found that the ProTherm database has numerous errors [18,26]. With such a high error rate, it is unimaginable that models built upon this database could achieve the accuracies as described in some of these papers. Taken together, it is necessary to evaluate the significance of the bias’s contribution to the performance issue of some programs for predicting protein stability change upon mutation.

This work is an attempt to examine whether models built on a combined dataset that included both forward and reverse mutations may suffer from the problem of data leakage. Data leakage refers to a situation when the training and testing datasets are overlapped or share significant similarity during the development process of ML models. Consequently, the performance evaluated on the test dataset can be over-estimated, resulting in unreliable and bad prediction outcomes in real-world applications. If this is true, the performance of these models is over-optimistic and the improvement by using HRMs to generate a balanced data may not be as significant as suggested by the bias theory advocators.

Data leakage happens when highly similar cases exist in training and test datasets. For protein stability change prediction, there may exist two types of data leakage: inter-protein and intra-protein ones. Intra-protein data leakage refers to the situation when mutations at the same location of a protein but with different incoming residues are split into training and test datasets. These mutations can be highly correlated. Inter-protein data leakage happens when mutations from two similar proteins are distributed to training and test datasets. Sequence similarity may exist locally even when two proteins have low overall similarity. In this study, a simple way was developed to construct a balanced dataset. It was then conjugated with a leave-one-protein-out approach (LOPO) that eliminates intra-protein data leakage. The study shows that it is highly possible data leakage may happen when HRMs are used in the training/testing and conventional n-fold cross validation (n-FCV) is employed [27]. Thus, data imbalance and the bias may only play a secondary role in the performance of the predictive models.

## Methods

The dataset used to develop I-Mutant2.0 [28] was chosen in this study because it was derived from the noisy ProTherm database and its 62 features are not sufficiently informative for predicting protein stability change upon mutation, as thoroughly analyzed previously [15]. Thus, models built based on these features and data aren’t expected to be robust and perform well.

The dataset for the training and test I-Mutant2.0 sequence-only SVM model was downloaded from https://folding.biofold.org/i-mutant//pages/dbMut.html. There are 2048 mutations from 64 proteins. Among them, 600 are stabilizing mutations, 31 are neutral, and 1417 are destabilizing mutations. To faithfully reproduce the results in literature, no attempt to reduce redundance was made as the literature [28]. The sequences of proteins in the dataset were retrieved from PDB and other relevant databases based on protein IDs available in the dataset. In addition to temperature and pH of the experiments available in the downloaded dataset, I-Mutant2.0 utilized 40 features calculated from the sequences. The first 20 values (for 20 residue types) encode the mutation by setting -1 to the residue corresponding to the deleted residue and 1 to the incoming residue, while all the remaining residues are set to 0. Each of the last 20 input values are the number of the encoded residue type inside a window of 19 residues centered at the mutation site.

### Balanced dataset

To demonstrate that the data imbalance is not the primary issue, a balanced dataset was constructed using a straightforward approach. A half of the entries in the forward dataset were randomly selected. The remaining half dataset was replaced with their corresponding reverse mutations. This dataset was named as “balanced” since the numbers of stabilizing and destabilizing mutations were almost identical. Since the number of replacement HRMs is identical to the replaced original mutations, the number of mutations in the balanced dataset is still 2048 from 64 proteins. Approximately half of these mutations are forward and the other half are hypothetical reverse mutations.

### Combined dataset

The forward and reverse datasets were merged into a combined dataset. Thus, there are 4096 cases in this dataset. This dataset is perfectly balanced. The combined approach was used by bias theory advocators to build their models.

### Leave-one-protein-out (LOPO)

A leave-one-protein-out approach was used in the study. Mutations from one single protein were used as test data while mutations from all other proteins were used to develop a model. Each protein was used as test data once. In this way, intra-protein data leakage is avoided. The results were then compared to 10-fold cross validation (10-FCV), an approach commonly used in the literature of the protein stability change upon mutation studies. In a conventional 10-FCV, all cases are randomly partitioned into 10 equal sized folds [27]. One of the folds is retained as the test dataset and remaining 9 folds are combined and used as training dataset. Therefore, some mutations from a protein are likely partitioned to training and test datasets, causing intra-protein data leakage.

Support vector machine (SVM) based predictive models, same as I-Mutant2.0, were built using the R e1071 package (https://cran.r-project.org/package=e1071).

### Performance metrics

Three statistical metrics were used to measure the performance of models in the study. The Pearson correlation coefficients of the experimental and predicted ΔΔG values were calculated. In addition, different ΔΔG values were used as thresholds to convert the predictions into binary classes (i.e., stabilizing and destabilizing), and then the area under receiver operating characteristic (ROC) curves were generated and the area under the ROC curve (AUC) were calculated. Finally, Q2, the proportion of the number of correct predictions to the number of examples, were calculated after the predictions were converted to binary classification, where mutation with negative ΔΔG values were considered destabilizing and positive ones as stabilizing [28].

## Results

**Fig 1:**
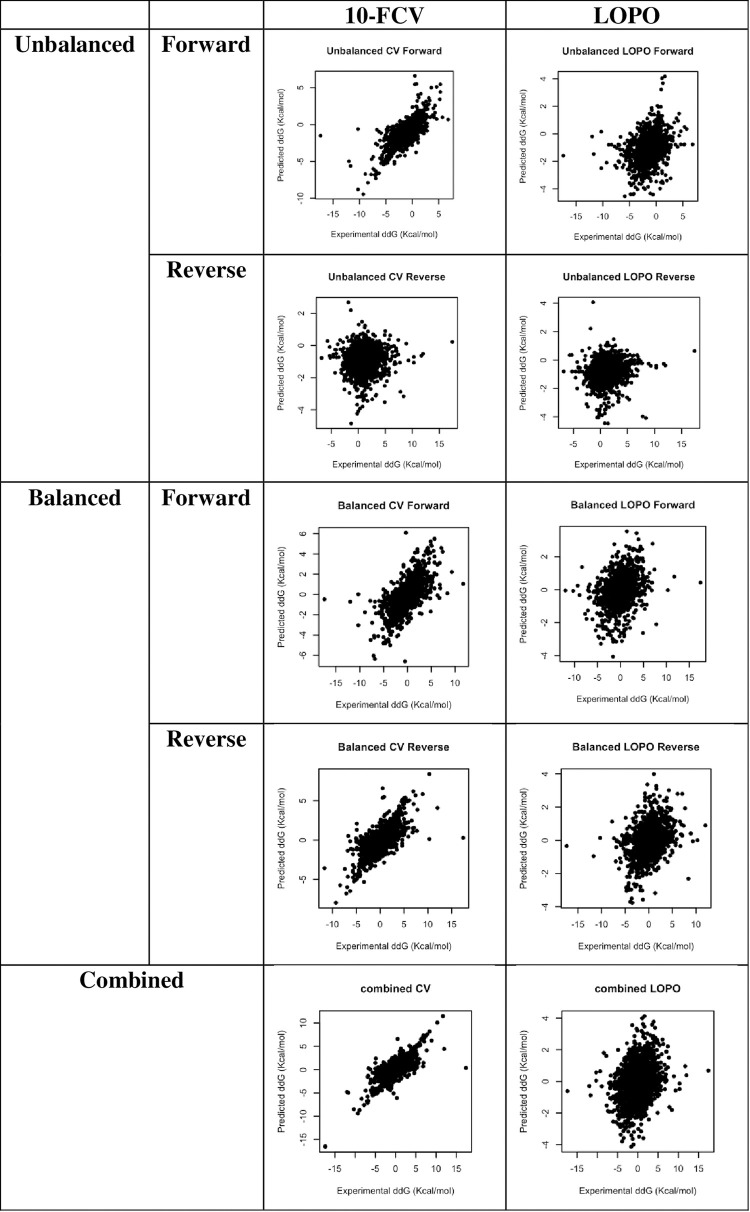
Scatter plots of experimental ΔΔG versus predictions of unbalanced, balanced, and combined approaches.

**Fig 2:**
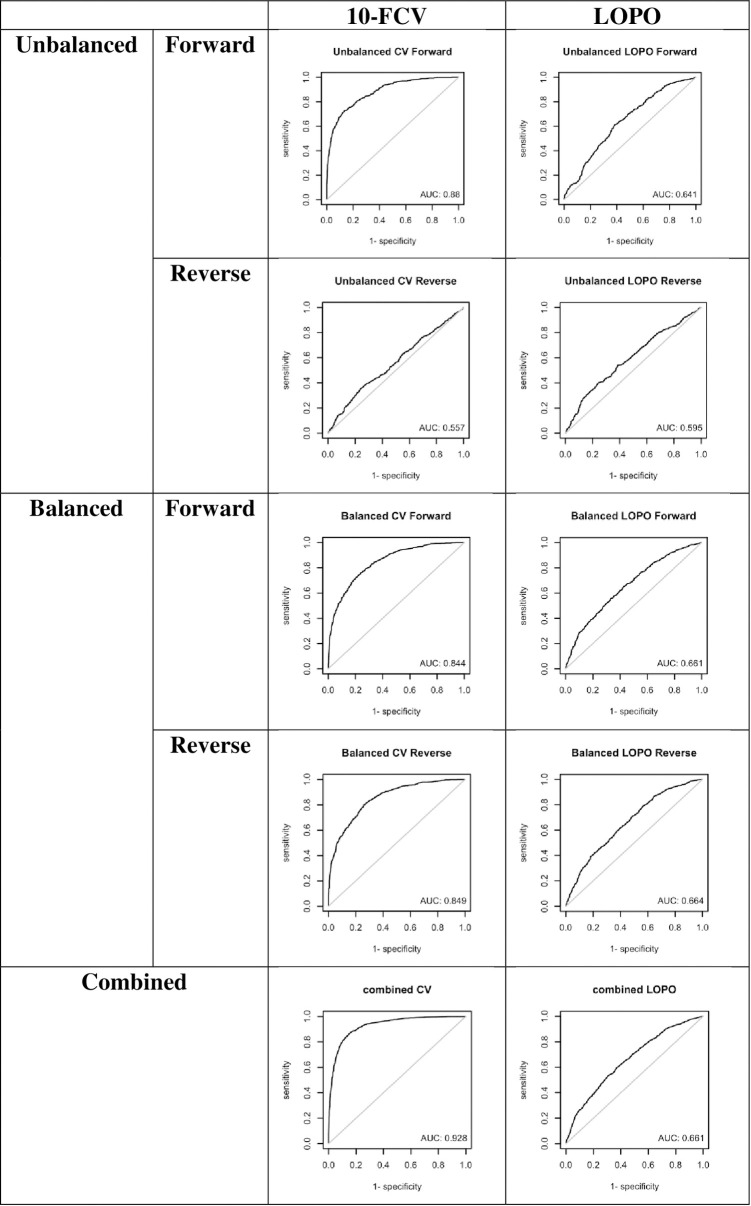
ROC curves and their AUCs of ΔΔG prediction of the unbalanced, balanced and combined approaches. Different ΔΔG values were used as thresholds to convert the prediction into binary stabilizing and destabilizing classes.

A series of experiments were performed to compare the LOPO and 10-FCV approaches (**Table 1**). The scatter plots and ROC curves of all experiments are provided in **Figs 1 and 2**. For the original unbalanced dataset, the predictions of forward mutations from 10-FCV have a Pearson Correlation Coefficient (R) of 0.7845 and area under curve (AUC) of 0.8803, similar to the results presented in the i-Mutant2 paper [28]. However, the prediction power largely diminished for the reverse mutations (R = 0.0523, AUC = 0.5569), revealing the data leakage problem of the model [15]. For the balanced dataset, the results are more consistent between forward and reverse mutations (R: 0.6727 and 0.7186, AUC: 0.8443 and 0.8494, respectively). For the combined dataset, the R and AUC (0.8705 and 0.9278, respectively) are better than the original unbalanced and balanced datasets, consistent with the results provided by the advocators of the bias theory but obviously over-optimistic, considering the model was based on noisy data and insufficiently informative features [15,22].

**Table 1:** Comparison of 10-FCV and LOPO results.

		10-FCV			LOPO		
		R	AUC	Q2	R	AUC	Q2
**Unbalanced**	Forward	0.7845	0.8803	0.8271	0.3336	0.6412	0.6851
	Reverse	0.0523	0.5569	0.3374	0.1326	0.5951	0.3545
**Balanced**	Forward	0.6727	0.8443	0.7514	0.3266	0.6608	0.5996
	Reverse	0.7186	0.8494	0.7432	0.3426	0.6643	0.6035
**Combined**		0.8705	0.9278	0.8474	0.3408	0.6606	0.6038

R: Pearson correlation coefficient; AUC: Area under ROC curve; Q2: Number of correct predictions/number of examples; 10-FCV: 10-fold cross validation; LOPO: Leave-one-protein-out.

The results of the LOPO experiments are more realistic than that of 10-FCV ones. The performance of the predictive model for the forward mutation is modest (R: 0.3336, AUC: 0.6412), notably different from the 10-FCV results (R: 0.7845, AUC: 0.8803). The predictions of the model on reverse mutations are even worse (R: 0.1326, AUC: 0.5951). This indicates that LOPO is more stringent than 10-CV and confirms that the data and features are not suitable for this type of prediction. For the balanced dataset, the performance of models on forward and reverse mutations are similar but have much lower R and AUC values than their corresponding experiments of the 10-CV approach. Since the data are noisy and the features are not sufficiently informative for protein stability changes, LOPO should be closer to the reality than 10-FCV. The combined dataset showed a very similar trend as the balanced dataset. Evidently, LOPO models built from all three datasets delivered similar results. Thus, the models of LOPO approach are likely more realistic than the 10-FCV approach and data imbalance does not play a significant role in prediction performance.

## Discussion and conclusions

The I-Mutant2.0 dataset was used in the study because it is among few if not the only dataset allowing other researchers calculate its features relatively easily. More importantly, these features have been thoroughly examined and deemed not possible to have significant prediction power [15]. Logically, if any model built upon this dataset and its features could achieve any meaningful predictive power, the tactic used to build such a model is questionable which should not be used as a proof of robustness.

Using the newly constructed balanced dataset, conjugated with the LOPO approach, the present study has demonstrated that data imbalance and therefore bias is not the primary reason that caused the performance issue, as models built using balanced and combined data were only able to achieve similar performance to the unbalanced data. If the bias plays a significant role, the performance of the balanced dataset should be better than the unbalanced one. But they are very similar in this study (R = 0.3266 for balanced vs. R = 0.3336 for unbalanced). More likely, the data and features are responsible for the unsatisfactory performance as we discussed previously **[15]**. While utilizing HRMs to deal with the bias is a good tactic that should improve performance when sufficient data and informatic features are used, it alone is unlikely sufficient to deal with fundamental issues such as poor-quality data and insufficiently informative features. HRMs should not be used to create balanced data AND evaluate the performance simultaneously, as they may cause the data leakage.

This work does not imply all the existing algorithms for predicting protein stability change upon mutation have the data leakage problem. Instead, the results suggest that solving data imbalance and consequently the bias issue alone doesn’t guarantee that the trained models are robust. Therefore, a balanced dataset with some seemly good conventional n-fold CV results should not be used as a proof that a model for predicting protein stability change upon mutations is robust. It is urgent to re-evaluate existing algorithms using more rigid approaches such as LOPO.

While it is true that LOPO is more stringent than 10-FCV in regarding of the problem under study, as it does not allow intra-protein data leakages. It should be pointed out, however, it is still possible that the LOPO may suffer from the inter-protein data leaking problem. Therefore, LOPO should be considered as a *necessary* but not *sufficient* proof of robustness of predictive models. Although the inter-protein data leakage problem was not addressed in the study, the LOPO study is adequate to illustrate the underlying performance problem of some of the algorithms developed for predicting protein stability change upon mutation.

In conclusion, this study provided strong evidences to support that the data and features, instead of data imbalance, may be the primary reason for the performance issue of some of the predictive models for protein stability changes upon mutations. Therefore, future research in the field should be focused on generating more significant amounts of reliable experimental data and informative features. Lessons learned from experimental results should be used to guide designing novel informative features [29]. It is encouraging that recently the ProTherm database was finally updated [30] and other new databases for protein stability changes have also been developed [19,26]. Besides, some newly developed algorithms used data with improved quality together with data balancing [31,32] while others introduced creative approach [33,34] and novel features [35]. Nevertheless, the models built upon the bias theory and estimated using conventional n-FCV, especially those using the old ProTherm database should be re-evaluated before any practical application. Future research in this field should be cautious of both inter- and intra- protein data leakages.

The present study also outlines the potential data leakage problem for applications of artificial intelligence (AI) including machine learning in medicinal chemistry and structural biology in general. Cautions should be always taken as the data leakage problem may happen subtly and the conventional cross validation approach may not provide realistic estimation.

## Supporting information

S1 Fig Scatter plots of experimental ΔΔG versus predictions. (DOCX)

S2 Fig ROC curves and their AUCs of ΔΔG prediction. (DOCX)

S1 File S2048. (XLS)

S2 File S2048_rev. (XLS)
