# Supplementary material for: The role of data imbalance bias in the prediction of protein stability change upon mutation
Source: PLoS One. 2023 Mar 30;18(3):e0283727. doi: 10.1371/journal.pone.0283727 (PMC10062539; doi:10.1371/journal.pone.0283727)
Supplement: S1 Fig — (DOCX) [file pone.0283727.s001.docx]

|  |  | **CV** | **LOPO** |
| --- | --- | --- | --- |
| **Unbalanced** | **Forward** | 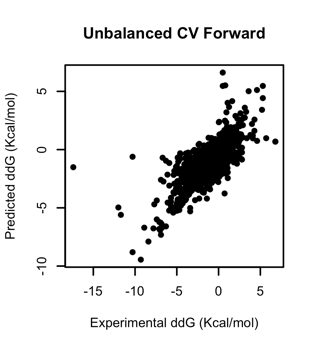 | 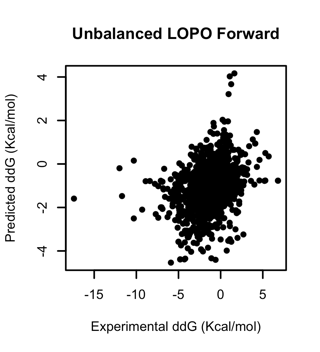 |
|  | **Reverse** | 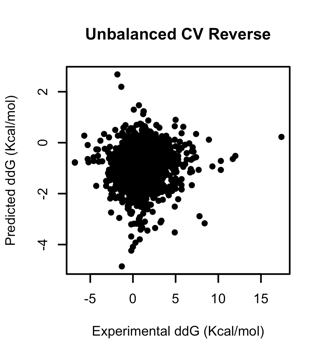 | 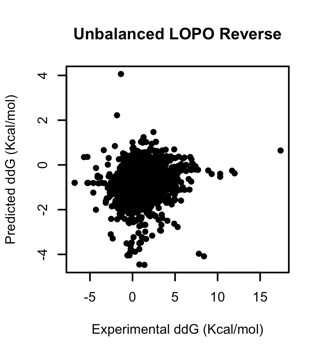 |
| **Balanced** | **Forward** | 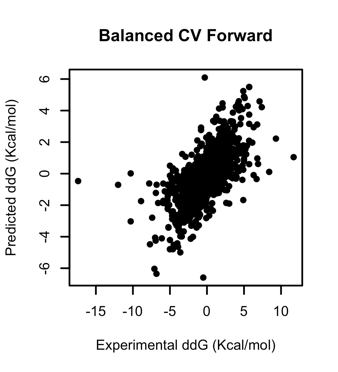 | 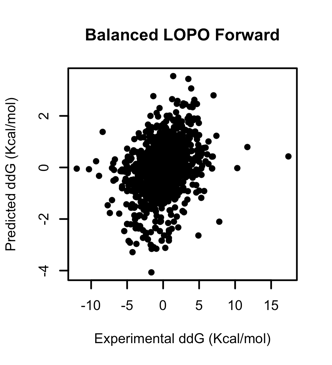 |
|  | **Reverse** | 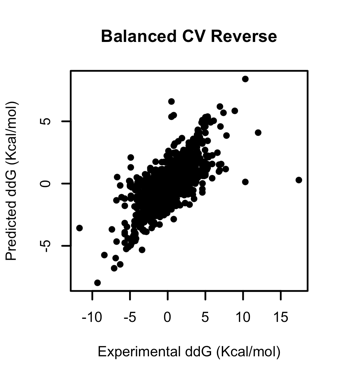 | 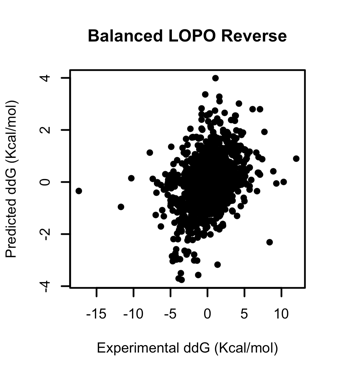 |
| **Combined** | | 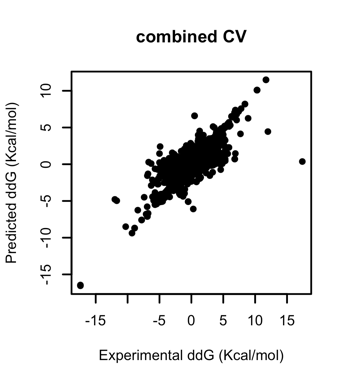 | 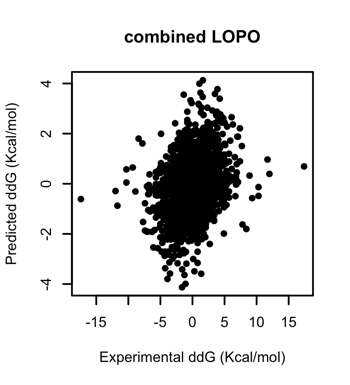 |

**Supplementary Figure 1**. Scatter plots of experimental ΔΔG versus predictions.

|  | | **CV** | **LOPO** |
| --- | --- | --- | --- |
| **Unbalanced** | **Forward** | 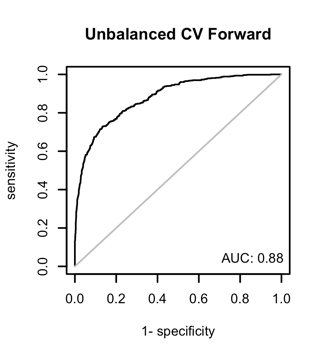 | 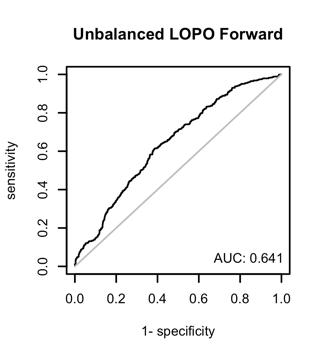 |
|  | **Reverse** | 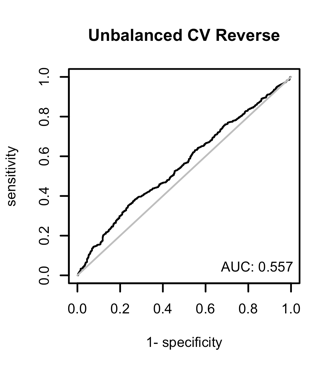 | 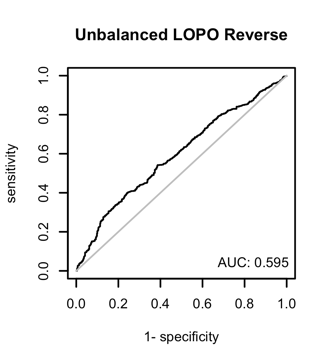 |
| **Balanced** | **Forward** | 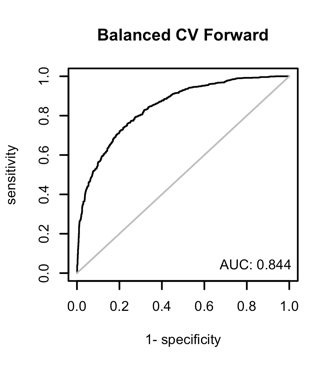 | 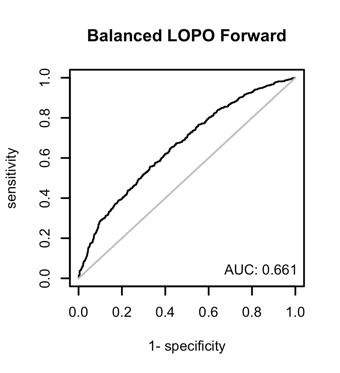 |
|  | **Reverse** | 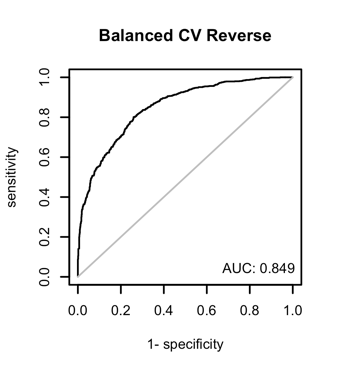 | 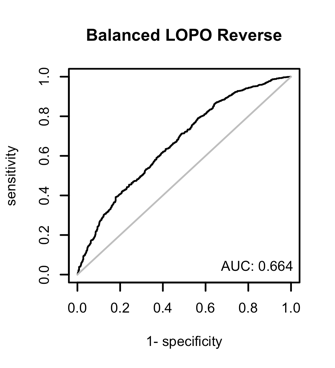 |
| **Combined** | | 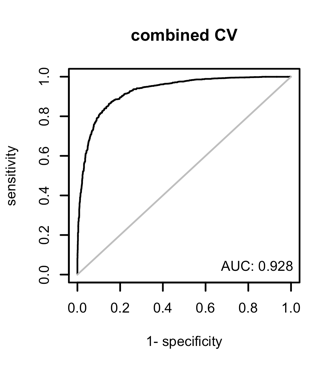 | 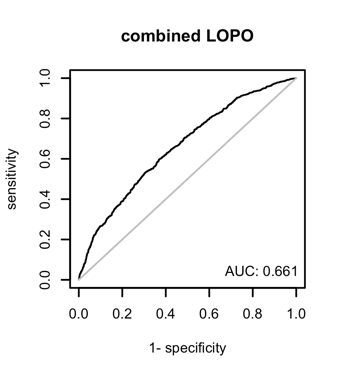 |

**Supplementary Figure 2**. ROC curves and their AUCs of ΔΔG prediction. Different ΔΔG values were used as thresholds to convert the prediction into binary stabilizing and destabilizing classes.
